# Prediction of Length of Hospital Stay of COVID-19 Patients Using Gradient Boosting Decision Tree

**DOI:** 10.1155/2022/6474883

**Published:** 2022-09-16

**Authors:** GholamReza Askari, Mohammad Hossein Rouhani, Mohammad Sattari

**Affiliations:** ^1^Department of Community Nutrition, School of Nutrition & Food Sciences, Isfahan University of Medical Sciences, Isfahan, Iran; ^2^Nutrition and Food Security Research Center, Isfahan University of Medical Sciences, Isfahan, Iran; ^3^Health Information Technology Research Center, Isfahan University of Medical Sciences, Isfahan, Iran

## Abstract

The aim of this paper is to predict the patient hospitalization time with coronavirus disease 2019 (COVID-19). It uses various data mining techniques, such as random forest. Many rules were derived by applying these techniques to the dataset. The extracted rules mainly were related to people over 55 years old. The rule with the most support states that if the person is between 70 and 80 years old, has cardiovascular disease, and the gender is female; then, the person will be hospitalized for at least five days. The gradient boosting random forest technique has performed better than other techniques. As a limitation of the study, it can be pointed out that a few features were unavailable and had not been recorded. Patients with diabetes, chronic respiratory problems, and cardiovascular diseases have a relatively long hospitalization. So, the hospital manager should consider a suitable priority for these patients. Older people were also more likely to take part in the selection rules.

## 1. Introduction

The coronavirus disease of 2019 (COVID-19) is a phenomenon that has plagued and killed many people in large numbers of countries [1]. COVID-19 is defined as a disease or infection by a new strain of coronavirus and it is called acute coronary syndrome. The devastating effects of COVID-19 are still being seen worldwide. These effects are also evident in the cultural and social dimensions. The disease spread rapidly and it has disrupted the ordinary lives of people. Moreover, it prevented people from attending many gatherings. Masks and social distance have been proposed as approaches to combat this disease. These approaches have led to dramatic changes in business conditions. Also, they have raised the new technologies issues [2, 3]. COVID-19 has many clinical features. The clinical features of COVID-19 vary from asymptomatic to severe disease and death [4]. Many underlying disorders, including cardiovascular disease, chronic kidney disease, chronic respiratory disease, diabetes mellitus (DM), hypertension, and obesity are represented as potential risk factors for severe COVID-19. The severe COVID-19 leads to hospitalization in the intensive care unit [ICU] and even death [5, 6]. COVID-19 is a multisymptom disease. The symptoms included fever, cough, fatigue, sputum production, diarrhea, and taste disturbances [7, 8]. Some patients also experienced muscle pain, fatigue, and loss of taste or smell [9]. Prolonged hospitalization has a high cost for the individual and the health system. It causes a significant burden, especially for the poor and low-income groups [10]. Prolonged hospitalization put a lot of pressure on hospitals and medical staff. Thus, it was challenging to manage the ICU beds in hospitals. Considering that the mortality rate of hospitalized patients varies from 5% to 25% [11], if the system can predict the patient hospitalization time, it will implement an effective strategy to overcome this issue. In fact, by indicating the hospitalization time, the managers can help make appropriate decisions about the allocation of hospital beds. It will also help improve decisions of the disease. The science of data mining has been proposed to reduce the workload of physicians. It provides a suitable model for making better decisions in recent years. The primary purpose of this paper is to predict the time of COVID-19 hospitalization by data mining techniques.

## 2. Materials and Methods

It consists of four parts: data collection, data preprocessing, modeling, and evaluation measures.

### 2.1. Data Collection

The database contains information of COVID-19 hospitalized patients. This information was available in the SIB system. The integrated health system (IHS) entitled “SIB” was launched in 2016 and aimed to act as an electronic health record (EHR) in the field of health. This system consists of more than 35,000 covid-19 patient information. Unfortunately, more than 14,000 of these people have died. As the dead people information is unrelated to hospitalization time prediction, only 21000 patient data were used. Also, the information about patients whose COVID-19 result is negative was ignored (*n* = 7000). So, it applies approximately 14000 patient data. Moreover, about 1700 patients were registered in ICU. This patient information was excluded from the dataset. So, the final evaluation applied on 12300 patient information. Database attributes include patient's age, gender, COVID-19 outcome, underlying diseases (cancer, chronic kidney disease, diabetes, cardiovascular disease, chronic neurological disease, AIDS, chronic blood disease, chronic liver disease, chronic respiratory disease, and hypertension), malnutrition, obesity, date of admission, sample date, date of discharge, sampling date, date of death, date of COVID-19 outcome, and pregnancy. The COVID-19 results consist of four values: negative, positive, repeated samples, and re-sampling. The underlying disease features were binary. The negative results were ignored.

### 2.2. Data Preprocessing

The first step in data preprocessing was to select a subset of related features. Most of the database features are unrelated. So, at first, the list of influential factors was determined using the opinion of cardiologists. Therefore, 19 out of 45 features were selected as the most relevant features of the dataset. Features that were unrelated to the study are removed from the attribute set. The removed features are sampling date, date of the COVID-19 outcome, and date of death. The date of determining the COVID-19 outcome is when the result of the COVID-19 tests is ready.

The age was divided into 18 to 55, 56 to 64, 65 to 69, 70 to 79, and 80 years and above. The hospitalization time is the target attribute, which is calculated by subtracting the date of discharge from the date of admission. Hospitalization time was divided into intervals of less than 24 hours [12], one to three days, four to five days, six to eight days, nine to ten days, and more than ten days. Some records miss the discharge date. As the admission time (target attribute) is calculated based on the discharge date, records whose discharge date was not defined were removed.

### 2.3. Modeling

Patients may have different hospitalization times, and the number of patients in each class will differ. For example, the class of one day has 1000 patients, and the class of more than 10 days has 40 patients. This difference will cause an imbalance in the number of patients in each class. So, the first step in modeling is using techniques to eliminate the imbalance in the dataset.

#### 2.3.1. Modeling-Imbalance in the Dataset

There are several ways to resolve the imbalance. One way is data balancing. Data balancing has two methods. The first one is random majority under-sampling, which balances class distribution by randomly deleting majority class instances. The second one is random minority oversampling (ROS), which adds randomly selected instances of the minority class (by replacement) to the original dataset.

#### 2.3.2. Modeling Techniques

The techniques used in this section include gradient boosting random forest, ID3, and random forest. Random forest [13] operates randomly by creating several trees at random and making decisions based on selection. The ID3 technique is a fuzzy decision tree-based with the most minor depth, in which each feature is placed in the tree growth path only once. This technique, unlike the random forest technique, is a definitive technique. The proposed method is the gradient boosting technique. This technique aim is creating a robust final model from a series of weak models.

### 2.4. Evaluation Measures

The evaluation part used different measures. One of these measures was accuracy [14]. The closer the accuracy to 1, the better the performance of the methods. This measure is calculated based on the following formula:

(1) $$\begin{array}{c} \text{Accuracy}=\frac{\text{TP}+\text{TN}}{\text{TP}+\text{TN}+\text{FP}+\text{FN}}, \end{array}$$

Another criterion is confidence [15] that the closer to one, the higher performance of the method.

## 3. Findings

According to Table 1, the number of COVID-19 patients with diabetes, the most common group of underlying diseases, was 1485. Cardiovascular disease is the second most common underlying disease among COVID-19 patients. The third most common disease is hypertension, which affects 1,201 COVID-19 patients. Moreover, about 200 COVID-19 patients were also battling cancer. The lowest number of people with underlying disease belonged to AIDS and splenectomy.  Table 2 represents values of gender, age, discharge, COVID-19 result, length of hospital stays, and pregnancy.

In Table 3, the random forest gradient boosting method performed better than the other techniques. It has acceptable performance in more than 73% of cases. This technique has also been able to improve the performance of the random forest by about 3.5%.

By applying different methods, 34 rules were derived. The proposed method chose the rules with more than 200 support. So, 9 out of 34 rules were derived, which are shown in Table 4.

The rule with the most support states that if the person is between 70 and 80 years old, has cardiovascular disease, and the gender is female; then, the person will be hospitalized for at least five days. The rule with the most confidence states that if the person is between 55 and 64 years old has a chronic kidney disease; then, the person will be hospitalized for between 1 and 5 days.

## 4. Discussion

This paper predicted the COVID-19 patients' hospitalization time by using various data mining techniques. Many rules were derived by implementing these techniques on the dataset. The extracts mainly were related to people over 55 years old. Diabetes, chronic respiratory disease, and cardiovascular disease patients have relatively long hospitalization. The rule with the most support states that if the person is between 70 and 80 years old, has cardiovascular disease, and the gender is female; then, the person will be hospitalized for at least five days. Older patients are more prone to COVID-19, which affects their hospitalization time [13]. The cardiovascular has less referring to hospital than before. So, their number of hospitalizations have decreased during the COVID-19 pandemic. The rule states with 83% confidence that if the person is between 55 and 64 years old, has chronic kidney disease, and the gender is male; then, the person will be hospitalized for between 1 and 5 days. Unlike people with heart disease, people with kidney diseases had more visits to medical centers during the COVID-19 period than before. Studies have also shown that kidney patients are more likely to develop COVID-19 coronary infections due to a weakened immune system [16]. The rule states with 71% confidence that if the person is between 55 and 64 years old, has diabetes, and the gender is male; then, the person has been hospitalized for more than 8 days. Also, another rule states with 81% certainty that if the person is between 18 and 54 years old and has diabetes; then, the person will be hospitalized for between 5 and 10 days. By examining these two rules and comparing them with other rules, the role of the underlying disease of diabetes is evident, so this disease has taken up the most hospitalization time. The interesting point in the rule with 72% confidence states that if the person is between 18 and 55 years old, has cancer, and the gender is male; then, the hospitalization time will between 1 and 3 days. The hospitalization time of cancer patients between 18 and 55 years is less of other patients. During the COVID-19 period, the number of cancer patients decreased compared to before this period [17]. The gradient boosting random forest technique has performed better than other techniques. As a limitation of the study, it can be pointed out that a few features were unavailable and had not been recorded.

## 5. Conclusion

This paper aimed to predict the hospitalization time of COVID-19 patients using decision tree-based techniques. The output of the article was in the form of rules. Diseases such as diabetes, chronic respiratory, and cardiovascular had more extended hospital stay than other diseases. So, the hospital manager should consider a suitable priority for these patients. Older people were also more likely to take part in the selection rules.

## Figures and Tables

**Table 1:** The number of COVID-19 patients with each underlying disease.

Underlying disease	The number of patients
Cancer	184
Cardiovascular disease	1105
Chronic liver disease	73
Chronic neurological disease	287
Chronic respiratory disease	478
Chronic kidney disease	326
Diabetes	1094
AIDS	4
Hypertension	1098
Obesity	65
Chronic blood disease	68
Other immunodeficiency diseases	54
Splenectomy	4

**Table 2:** Lists of the other attributes and their different values.

Attributes	Type of values
Pregnancy	Yes-No

Discharge	Outpatient
	Hospitalization

Age	<18
	18–55
	56–64
	65–69
	70–79
	≥80

Gender	Female-male

COVID-19 result	Negative
	Positive
	Positive again
	Need for re-sampling

Length of hospital stay	<1 day
	1–3 days
	4–5 days
	6–8 days
	9–10 days
	>10 day

**Table 3:** The accuracy of different data mining techniques.

Techniques	Accuracy (%)
ID3	72.28
Random forest	70.13
Gradient boosting random forest	73.51

**Table 4:** Extracted selected rules regarding the prediction of the hospitalization time of COVID-19 patients.

Extracted selected rules	Support	Confidence (%)
If the person is between 18 and 55 years old, has cancer, the gender is male, and the COVID-19 result is positive; then, the person will be hospitalized for between 1 and 3 days	285	72
If the person is between 70 and 80 years old, has a cardiovascular disease, and the gender is female; then, the person will be hospitalized for at least 5 days	2570	76
If the person is between 55 and 64 years old, has a chronic kidney disease, and the gender is male; then, the person will be hospitalized for between 1 and 5 days	874	83
If the person is between 18 and 54 years old and has a chronic liver disease; then, the person will be hospitalized for 4 to 5 days	547	70
If the person is over 65 years old, has a chronic neurological disease, and the gender is female; then, the person will be hospitalized for between 1 and 5 days	475	75
If the person is between 65 and 70 old, has a chronic respiratory disease, and the gender is male; then, the person will be hospitalized for between 1 and 8 days	319	68
If the person is between 18 and 54 years old and has diabetes; then, the person will be hospitalized for between 5 and 10 days	727	81
If the person is between 55 and 64 years old, has diabetes, and the gender is male; then, the person will be hospitalized for more than 8 days	673	71
If the person is a woman, over 80 years old, and has hypertension; then, the person will be hospitalized for less than five days	823	69

## Data Availability

The used datasets are not freely available. It consists of COVID-19 patients of Alzahra Hospital in Iran.
